# An eco‐evolutionary feedback loop between population dynamics and fighter expression affects the evolution of alternative reproductive tactics

**DOI:** 10.1111/1365-2656.12899

**Published:** 2018-09-19

**Authors:** Jasper C. Croll, Martijn Egas, Isabel M. Smallegange

**Affiliations:** ^1^ Institute for Biodiversity and Ecosystem Dynamics University of Amsterdam Amsterdam The Netherlands

**Keywords:** adaptive dynamics, alternative reproductive phenotype, cannibalism, coexistence, environmental threshold model, population dynamics

## Abstract

1. Surprisingly, little is known about how eco‐evolutionary feedback loops affect trait dynamics within a single population. Polymorphisms of discrete alternative phenotypes present ideal test beds to investigate this, as the alternative phenotypes typically exhibit contrasting demographic rates mediated through frequency or density dependence, and are thus differentially affected by selection.
2. Alternative reproductive tactics (ARTs), like male fighters and sneakers, are an extreme form of discrete phenotype expression and occur across many taxa. Fighters possess weapons for male–male competition over access to mates, whereas sneakers are defenceless but adopt tactics like female‐mimicking. Because fighters in some species mortally injure conspecifics, this raises the question whether fighter expression can feed back to affect population size and structure, thereby altering the selection gradient and evolutionary dynamics of ART expression in an eco‐evolutionary feedback loop.
3. Here, we investigated how the eco‐evolutionary feedback loop between fighter expression and population size and structure affects the evolution and maintenance of ARTs. We introduced intraspecific killing by fighters in a two‐sex, two‐ART population model parameterized for the male dimorphic bulb mite (*Rhizoglyphus robini*) that includes life‐history differences between the ARTs and a mating‐probability matrix analogous to the classic hawk–dove game.
4. Using adaptive dynamics, we found that the intraspecific killing by fighters can extend the range of life‐history parameter values under which ARTs evolve, because fighters that kill other fighters decrease fighter fitness. This effect can be nullified when benefits from killing are incorporated, like increased reproduction through increased energy uptake.
5. The eco‐evolutionary feedback effects found here for a dimorphic trait likely also occur in other fitness‐related traits, such as behavioural syndromes, parental care and niche construction traits. Current theoretical advances to model eco‐evolutionary processes, and empirical steps towards unravelling the underlying drivers, pave the way for understanding how selection affects trait evolution in an eco‐evolutionary feedback loop.

## Graphical abstract

Life cycle links between killing and copulating highlight the importance of studying eco‐evolutionary interactions. Using a theoretical model, the authors show how the intraspecific killing of conspecifics affects the ecological dynamics of a population, which feeds back to influence the evolution of what reproductive strategies males employ to gain access to females.

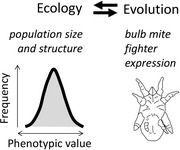

## 1 INTRODUCTION

Understanding the mechanisms of how a phenotypic character distribution of individuals within a population changes over time is a first step towards understanding how the joint dynamics of ecological and evolutionary processes affect populations (Smallegange & Coulson, 2013), of which we still know remarkably little (Hendry, 2016). One type of characters, or phenotypes, that are particularly likely to be influenced by both ecological and evolutionary processes are polymorphisms of discrete alternative phenotypes; examples of which include mating phenotypes in males (major vs. minor), protective phenotypes (armed vs. unarmed) or life cycle phenotypes (single vs. multiple reproductive bouts) (Oliveira, Taborsky, & Brockmann, 2008). The alternative phenotypes typically exhibit contrasting demographic rates: For example, mating and life cycle phenotypes differ in reproductive strategy and output, growth and even survival rates. When the contrasting demographic rates of alternative phenotypes are mediated through frequency and/or density dependence (Oliveira et al., 2008), which can be differentially affected by selection (Sinervo, Svensson, & Comendant, 2000), eco‐evolutionary feedbacks are likely to occur. Alternative reproductive tactics (ARTs) are extreme forms of such phenotypic variation within single populations, and arise over evolutionary time when there is high competition for mates (Oliveira et al., 2008). Typically, ARTs occur in one of the sexes, often the male, and are discrete phenotypes with distinct mating tactics (Oliveira et al., 2008). There are usually two phenotypes: fighters and sneakers. In many male dimorphic species, male morph expression is a conditional strategy (Oliveira et al., 2008), so that the specific (permanent) morph a male develops into depends on its condition during a critical point in ontogeny. Whereas the environment to a large extent determines the condition of a male, the threshold response of what morph a male of a certain condition develops into, is in many species under polygenic control and heritable (Oliveira et al., 2008). Fighters typically are large, may mature slowly and possess weapons that are used to obtain and guard mates, whereas sneakers are small and have no weapons, and resort to alternative methods of gaining access to females such as circumventing fighters or maturing early, and consequently mate earlier in life than fighters from the same age cohort.

**Figure 1:**
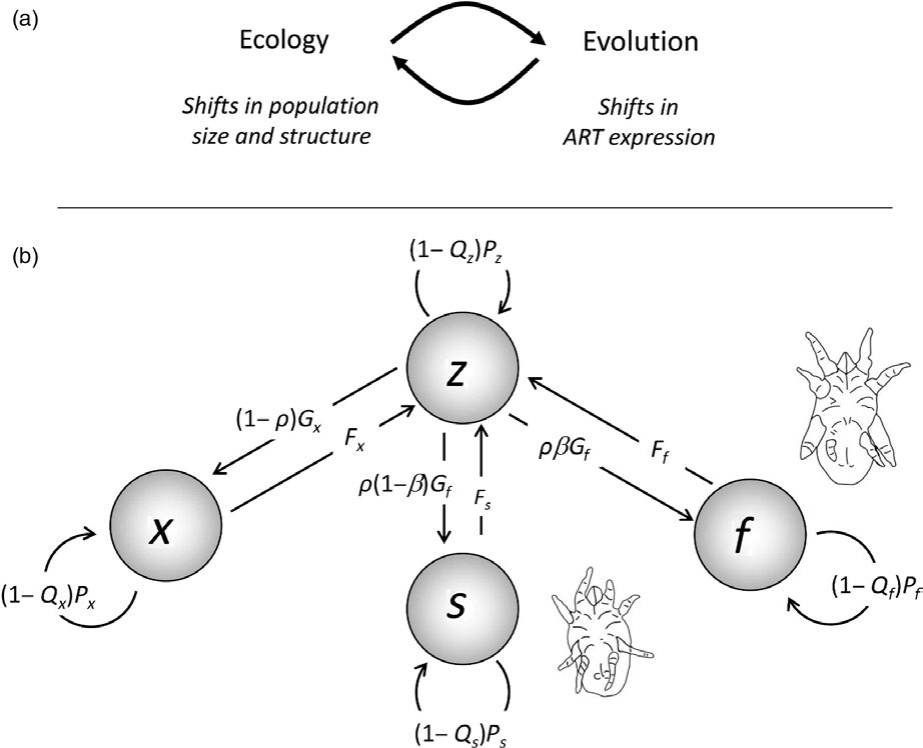
(a) The eco‐evolutionary feedback loop of Alternative reproductive tactics (ART) expression where shifts in population size and structure affect the selection gradient of ART expression (ecology‐to‐evolution pathway), and where evolutionary shifts in ART expression affect population size and structure (evolution‐to‐ecology pathway). (b) Stylized life cycle of a male dimorphic species in which fighters can kill conspecifics. Females (*x*), adult fighters (*f*) and adult scramblers (*s*) produce zygotes (*z*) at rates *F*_*i*_ (*i *= *x*,* f*,* s*). Zygotes develop into females, fighters or scramblers at rates that are set by the sex ratio (*ρ*) and male morph ratio (*β*). Terms *P*_*i*_ and *G*_*i*_ (*i *= *z*,* x*,* f*,* s*) denote survival and growth rates, respectively. In this life cycle, fighters can kill individuals of each life stage, which reduces the survival rate *Pi* (*i *= *z*,* x*,* f*,* s*) of each life stage by a proportion (1 − *Q*_*i*_) (see Equation (7) in main text)

Currently, the environmental threshold (ET) model is the leading theory to explain the evolution and maintenance of conditionally expressed ARTs (Hazel, Smock, & Johnson, 1990; Hazel, Smock, & Lively, 2004). The ET model is based on the premise that ART expression depends on whether or not an individual reaches a critical threshold, or switch point, during ontogeny. This threshold is assumed to be based on a continuously distributed, polygenic trait, called the “liability,” which can be a hormone profile. ART expression occurs in response to a cue (such as body size) that reliably informs on an individual's status within the mating environment. Individuals with a cue value above the threshold express one phenotype, while those below the threshold express the alternative. The ET model assumes that, in response to environment‐specific individual‐level selection, ARTs have evolved different fitness functions, through which selection can affect the distribution of individual liabilities. Because ART frequency depends on the distribution of individual liabilities and the cue distribution, both are taken into account in determining how ART fitness influences the evolution of liabilities and hence the evolution of the threshold. Predictions from the ET model on the evolution of the threshold, and thereby ART expression, have been tested successfully in experimental evolution studies (Tomkins, Hazel, Penrose, Radwan, & LeBas, 2011). However, when one such experimental study was repeated, but this time allowing for population feedback by keeping generations overlapping when applying selective mortality regimes (as opposed to taking a non‐random sample of individuals of the current generation to start the next), some evolutionary responses to selective mortality were diametrically opposite to the predictions from the ET model (Smallegange & Deere, 2014). If we understand why this mismatch occurred, we would be one step closer to understanding how the joint dynamics of ecological and evolutionary processes, and their interactions, affect population size, growth and persistence. A logical way forward is therefore to investigate how the ecological dynamics of shifts in population size and structure affect the evolution of ART expression by altering the selection gradient, that is the ecology‐to‐evolution pathway, and how evolutionary change in ART expression in turn affects population size and structure, that is the evolution‐to‐ecology pathway, within an eco‐evolutionary feedback loop (Figure 1a).

In some species, fighters mortally injure conspecifics (Fox, 1975). This raises the question whether fighter expression can feed back to affect population size and structure, thereby altering the selection gradient and evolutionary dynamics of ART expression in an eco‐evolutionary feedback loop. In this study, therefore, we explored to what extent the evolution and maintenance of ARTs is affected by such eco‐evolutionary interaction between evolution of fighter expression and (ecological) change in population size and structure. To do so, we employed the adaptive dynamics framework (Geritz et al., 1998; Metz, Nisbet, & Geritz, 1992), which has been instrumental in understanding how eco‐evolutionary feedback influences the evolutionary trajectories of trait dynamics (e.g. Lion, 2017). Because adaptive dynamics is built on the premise that trait evolution is the result of invasion by rare mutants (and not of short‐term shifts in genotype frequencies), the eco‐evolutionary feedback loop (Figure 1a) is played out at different time‐scales: There is a temporal sequence in which ART expression evolves, followed by equilibration of population size and structure, followed by ART evolution, and so on, until an ESS of ART expression is reached. We modelled the population dynamics using an existing two‐sex, two‐ART demographic model, which comprises the demographic rates of adult females, fighters, sneakers and their offspring, zygotes (Figure 1b). The proportion of male zygotes that develops into fighters is denoted by *β* (Figure 1b); thus, 0 < *β* < 1 represents male dimorphism. The parameter *β* can be interpreted as being determined by the threshold of ART expression in a conditional strategy analogous to the ET model (Smallegange & Johansson, 2014), which can thus evolve in response to selection (Hazel et al., 1990, 2004). Specifically, we aimed to understand (a) how the probability *u*_*i*_ that an individual of a targeted life stage *i* is killed when encountered by a fighter (which determines the proportion *Q* of the targeted life stage that is killed by fighters: Figure 1b) affects the evolutionarily stable strategy (ESS) of *β*, (b) how the probabilities that individuals of two different, targeted life stages are killed when encountered by fighters interactively influences this ESS, and (c) how intraspecific killing affects the effect of survival of the male morphs and competition parameters on this ESS. Intraspecific killing by fighters has mainly been linked to the killing of competitor males in conflicts over access to mates; the intraspecific killing of juveniles and females can also occur for other reasons, such as the early elimination of competition, or as an additional food source (Fox, 1975). Regardless of why fighters kill conspecifics, it results in increased mortality of the life stage targeted by the killing fighters, and it is probable that the demographic consequences of this intraspecific killing can affect the evolution of ARTs through the reduced survival of the targeted life stage (ecology‐to‐evolution pathway) and that the evolutionary trajectory of ART expression can in turn result in changes in population structure (the evolution‐to‐ecology pathway) (Figure 1a). We also varied the competition parameters probability of engaging in competition, sneaker advantage, fighter costs and reward, as these have been shown to influence the evolution and maintenance of ARTs (Smallegange & Johansson, 2014). By including these parameters in our analyses, we could assess whether interactive effects between intraspecific killing and any of these parameters on the evolution of male morph coexistence occur. In addition, we explored how variation in survival affects the evolution and maintenance of ARTs in response to variation in intraspecific killing, in order to assess whether the effect of intraspecific killing depends on background mortality rates. For our study, we extended the two‐sex, two‐ART population model of Smallegange and Johansson (2014) by including intraspecific killing by fighters, and parameterized the model for bulb mites (*Rhizoglyphus robini*). Sneaker male bulb mites are known as scramblers, and, in our model analyses, we therefore refer to males as either being a fighter or a scrambler.

## 2 MODEL

### 2.1 Baseline structure of the population model

The two‐sex, two‐ART population model is based on a stylized life cycle of a male dimorphic species, and consists of four life stages (Figure 1b) (Smallegange & Johansson, 2014): zygotes (*z*), adult females (*x*), fighters (*f*) and scramblers (*s*). Their population densities are denoted *n*_*i*_ (*i* = *z*,* x*,* f*,* s*). Zygotes are undifferentiated with respect to sex or male morph. The proportion of zygotes that develops into males equals *ρ*, and the proportion of males that develops into fighters equals *β*. A biological mechanism underlying the fraction *β* can be found in the ET model (Hazel et al., 1990, 2004). The development into an ART is determined by whether an ontogenetic cue (such as body size) exceeds a threshold value with a polygenetic basis at a certain point during development. In this case, *β* represent the fraction of individuals in which the cue exceeds the threshold and therefore mature into a fighter. An increase of *β*, given a certain regime of environmental variability, thus directly indicates a decrease in the threshold value and vice versa (Smallegange & Johansson, 2014). The rate (individuals per time step) at which zygotes of a certain life stage mature into the adult stage is denoted *G*_*i*_ (*i *= *x*,* f*,* s*) and calculated as $G_{j}\;=\;\sigma_{\mathrm{zz}}/t_{i}$, with $\sigma_{\mathrm{zz}}$ as the survival rate of zygotes and *t*_*i*_ as the maturation time of zygotes maturing in the adult stage *i*. Hence, the rate at which zygotes enter the female, fighter and scrambler adult stages equals (1 − ρ)*G*_*x*_, *β* ρ *G*_*f*_ and (1 − *β*)ρ *G*_*s*_, respectively. The rate (individuals per time step) of surviving and staying in a stage equals *P*_*i*_ (*i *= *z*,* x*,* f*,* s*), which, for adults, is calculated as *P*_*j*_ = $\sigma_{\mathrm{jj}}$, with $\sigma_{\mathrm{jj}}$ as the survival rate of morph *j* (*i *= *x*,* f*,* s*), and for zygotes as the following:

(1) $$P_{z}\;=\;\sigma_{\mathrm{zz}}\left(1-\left[\left(1-\rho \right)G_{x}\;+\;\beta \rho G_{f}\;+\;\rho \left(1-\beta \right)G_{s}\right]\right)$$

The fertility functions of females and males of morph *j* (*j *= *f*,* s*) are given by $F_{x}\;=\;\left(B_{x,s}\;+\;B_{x,f}\right)/\left(2n_{x}\right)$ and $F_{j}\;=\;1/\left(2n_{x}\right)B_{x,j}$, respectively, which both depend on the number of births, *B*_*x,j*,_ resulting from matings between females and males of morph *j*. The number of births from matings between females and morph *j* is defined as $B_{x,j}\;=\;ke_{x,j}p_{x,j}$, and depends on (a) the clutch size (*k*), which decreases with female density, $k\;=\;k_{0}/\left(1\;+\;n_{x}\right)$, (b) the encounter rate between a male of morph *j* and a female $e_{x,j}\;=\;e_{0}n_{x}n_{j}/\left(n_{x}\;+\;n_{s}\;+\;n_{f}\right)$, with *e*_*0*_ as the number of individuals of any life stage encountered by the focal individual per time step, and (c) the probability that an encounter results in a successful mating (*p*_*x,j*_). The probability that an encounter results in a successful mating depends upon the strength of male–male competition (*c*_*m*_), and the probability that a male of morph *j* gains access to a female when competing with a male of morph *j* is given by the following hawk–dove gamelike payoff matrix:

(2) $$M\;=\;\left[\begin{array}{cc} m_{f\;f} & m_{\mathrm{fs}}\\ m_{\mathrm{sf}} & m_{\mathrm{ss}} \end{array}\right]\;=\;\left[\begin{array}{cc} (V-C)/2 & V-\varepsilon \\ \varepsilon & V/2 \end{array}\right]$$

In this matrix, *V* is the probability of accessing a female without costs, *C* are the fighter costs in terms of the probability of gaining access to a female, and *ε* is the probability of sneaking successfully. We assume *ε* < *V*. This results in the probability that an encounter leads to a successful mating:

(3) $$p_{x,j}\;=\;1-c_{m}\;+\;c_{m}\left(\frac{n_{f}}{n_{f}\;+\;n_{s}}m_{\mathrm{jf}}\;+\;\frac{n_{s}}{n_{f}\;+\;n_{s}}m_{\mathrm{js}}\right)$$

The resulting population projection matrix includes all of the demographic rates of the four stage classes:

(4) $$A\;=\;\left[\begin{array}{cccc} P_{z} & F_{x} & F_{f} & F_{s}\\ \left(1-\rho \right)G_{x} & P_{x} & 0 & 0\\ \rho \beta G_{f} & 0 & P_{f} & 0\\ \rho \left(1-\beta \right)G_{s} & 0 & 0 & P_{s} \end{array}\right]$$

The two‐sex, two‐ART population model is defined as *n*_*t*+1_ = **A**_*t*_ *n*_*t*_, where *n*_*t*_ is the population vector at time *t* and **A**_*t*_ is the projection matrix **A** at time *t* that is determined by the population vector at time *t*.

### 2.2 Adjusting the baseline structure: including intraspecific killing

In the baseline structure of the model, encounter rates are calculated using only the densities of the adult stages (females, fighters and scramblers). Because we use this model to also investigate the effect of intraspecific killing on zygotes (see below), the encounter rate with zygotes has to be included. To achieve this, we calculated the encounter rate between individuals of stage *j* and individuals of stage *i* as:

(5) $$e_{i,j}\;=\;e_{0}\frac{n_{i}n_{j}}{\sum n_{i}}\;=\;e_{0}\frac{n_{i}n_{j}}{n_{z}\;+\;n_{x}\;+\;n_{f}\;+\;n_{s}}$$

The focus of this study was to investigate the effect of intraspecific killing on the evolution of *β*, which is the fraction of male zygotes that mature into fighters. Let *u*_*i*_ be the probability that an individual of a targeted life stage *i* (*i *= *z*,* x*,* f*,* s*) is killed when encountered by a fighter. We refer to the probability *u*_*i*_ as the killing success, which ranges from zero to one. When *u*_*i*_ equals zero, encountered individuals of the targeted life stage are not killed by fighters, and when *u*_*i*_ equals one, all individuals from the targeted life stage are killed when encountered by a fighter. The number of encounters per time step between the targeted life stage and fighters (*e*_*i,f*_) is calculated using Equation (5). The number of individuals within the targeted life stage killed per time step by fighter males (*q*_*i*_) equals:

(6) $$q_{i}\;=\;e_{i,f}u_{i}$$

The number of individuals within the targeted life stage killed per time step by fighters (*q*_*i*_) is used to calculate the proportion of the targeted life stage that is killed per time step by fighters (*Q*_*i*_), which can also be interpreted as the probability that an individual from the targeted life stage is killed by a fighter in the current time step:

(7) $$Q_{i}\;=\;\frac{q_{i}}{n_{i}}\;=\;e_{0}\frac{u_{i}n_{j}}{n_{t}\;+\;n_{x}\;+\;n_{f}\;+\;n_{s}}$$

Intraspecific killing is incorporated into the model by multiplying the survival rate without intraspecific killing (*P*_*i*_) (default survival rate) with the proportion of individuals of the targeted morph that is not killed by fighter males (*1* − *Q*_*i*_). This means that we assume that individuals that mature, that is move from the zygote to an adult stage, are not vulnerable to intraspecific killing. This is probable, because during maturation, *R. robini* enters an immobile, quiescent stage during which individuals hide, and we assume that it is unlikely that these individuals are attacked by fighters. Table 1 gives an overview of all of the parameters and parameter values used in the model.

**Table 1:** Parameter definitions and values

Parameter	Definition	Value	Range when varied^a^	Unit
*e*_0_	Number of individuals encountered by focal individual per time step	1		ind/day
Maturation				
*β*	Proportion of male zygotes developing into a fighter	–	0.00–1.00	–
*ρ*	Proportion of individuals maturing into females	0.5		–
*t*_*x*_	Female maturation time	13.7		days
*t*_*f*_	Fighter maturation time	14.4		days
*t*_*s*_	Scrambler maturation time	12.6		days
Competition and reproduction				
*c*_*m*_	Strength of male–male competition	0.9	0.00–1.00	–
*C*	Costs for a fighter of fighting another fighter	0.7	0.50–1.00	–
*V*	Probability of accessing a female without costs	1	0.50–1.00	–
*ε*	Probability of sneaking successfully by a scrambler when opponent is a fighter	0.2	0.00–0.40	–
*k*_*0*_	Density‐independent clutch size per mating	26.1		ind/mating
Survival				
*σ*_*zz*_	Zygote survival rate	1		day^−1^
*σ*_*xx*_	Female survival rate	0.95		day^−1^
*σ*_*ff*_	Fighter survival rate	0.95	0.90–1.00	day^−1^
*σ*_*ff*_	Scrambler survival rate	0.96	0.90–1.00	day^−1^
Intraspecific killing				
*u*_*z*_	Probability that a zygote is killed when encountered by a fighter	0	0.00–1.00	–
*u*_*x*_	Probability that a female is killed when encountered by a fighter	0	0.00–1.00	–
*u*_*f*_	Probability that a fighter is killed when encountered by a fighter	0	0.00–1.00	–
*u*_*s*_	Probability that a scrambler is killed when encountered by a fighter	0	0.00–1.00	–

a When a parameter was varied in one of the analyses, the range in which the parameter was varied is also given. All values, except for those under “*Intraspecific killing,*” are taken from Smallegange and Johansson (2014).

### 2.3 Population model

Based on all of the demographic rates and intraspecific killings, we derive the following population projection matrix **N**:

(8) $$N\;=\;\left[\begin{array}{cccc} \left(1-Q_{z}\right)P_{z} & F_{x} & F_{f} & F_{s}\\ \left(1-\rho \right)G_{x} & \left(1-Q_{x}\right)P_{x} & 0 & 0\\ \rho \beta G_{f} & 0 & \left(1-Q_{f}\right)P_{f} & 0\\ \rho \left(1-\beta \right)G_{s} & 0 & 0 & \left(1-Q_{s}\right)P_{s} \end{array}\right]$$

The population model is defined as *n*_*t*+1_ = **N**_*t*_ *n*_*t*_, where *n*_*t*_ is the population vector at time *t* and **N**_*t*_ is the projection matrix at time *t* (Equation (8)) that is determined by the population vector at time *t*.

### 2.4 Evolutionary dynamics

The adaptive dynamics approach (Geritz et al., 1998) was used to find the evolutionarily singular strategy of *β*. This approach assumes that the resident population with trait value *β* is in population‐dynamic equilibrium when a new mutant appears. Whether mutants with trait value *β*’ will successfully invade the resident population with trait value *β* depends on their initial population growth rate, or invasion fitness, in the context of a resident population in equilibrium, *W*(*β’, β*). Population growth rates are calculated by taking the dominant eigenvalue of the matrix **N** (Equation (8)); for the resident population in equilibrium, *W(β, β*) is always equal to one. The invasion fitness of the mutant is calculated by taking the dominant eigenvector of matrix **N’**, which is matrix **N** in which the values of *β* are replaced with *β’*, while all other variables are kept equal to the values in matrix **N** under the population‐dynamic equilibrium reached with the value of *β* (cf. Kisdi, 2002). This means that if the dominant eigenvalue of the matrix **N’** is greater than one, the mutant trait *β*’ will invade the population. We calculated the invasion fitness for all combinations of values of *β*’ and *β* between 0 and 1 in steps of 0.01. The characteristic equation of **N’** can be solved if one assumes that different morphs and sexes differ only in their growth rates (see Appendix of Smallegange & Johansson, 2014). However, in this study we took a more system‐specific approach (without losing sight of the more general questions) and incorporated differences in survival rates between the morphs and sexes (Equation (8)). For this reason, we were unable to solve the model analytically, and instead, we ran simulations. To this end, we first used the population model *n*_*t*+1_ = **N**_*t*_ *n*_*t*_ with the resident trait value *β* to create a time series of 1,000 time steps *t*, in order to arrive at the equilibrium densities at *t *=* *1,000. Whether equilibrium was reached was verified by checking whether the dominant eigenvalue was equal to one. We then replaced the resident trait value of *β* with the mutant value *β*’ and calculated the dominant eigenvalue of the resulting matrix **N’** to arrive at the invasion fitness. Therefore, it is assumed that the invading mutant experiences the population structure of the resident population.

The direction of evolutionary change is determined by the selection gradient, which is defined as the slope of the invasion fitness with regard to the variant trait at *β*’ = *β*. When the selection gradient is positive (negative), a mutant with a slightly higher (lower) trait value can invade the population and replace the resident. In adaptive dynamics, a candidate evolutionary endpoint can be found when the selection gradient equals zero. When this point is also resistant to invasion and is an attractor for gradual evolution, it can be considered an ESS (McGill & Brown, 2007). We assessed these ESS criteria using pairwise invasibility plots (PIPs). The invasion fitness is always equal to zero when *β*’ = *β* and represents the primary isocline in the PIP. Situations in which the invasion fitness is zero while *β*’ ≠ *β* represents the secondary isocline. The intersects between the primary isocline and the secondary isocline represent candidate evolutionary endpoints (β*). We assessed whether each candidate evolutionary endpoint was convergence and evolutionarily stable using three visual criteria (Geritz et al., 1998): (a) if the vertical line through β* is entirely in a negative invasion fitness area, this indicates that β* cannot be invaded, and is therefore evolutionarily stable; if this is not the case, β* is an evolutionary repeller, and the value of *β* will evolve away from this value; (b) any resident strategy can be invaded by a mutant closer to the singular strategy, β*, if the area to the left of the secondary isocline and immediately above the primary isocline and the area to the right of the secondary isocline and below the primary isocline are positive invasion fitness areas; in this case, β* is convergence‐stable; (c) if the horizontal line through β* is entirely within a positive invasion fitness area, this indicates that β* can always spread through the population when initially rare. When all of these criteria are met, *β*’ can be considered an ESS (*β*_ESS_). In some cases, the singular strategy is an evolutionary repeller (see also Results); in all other cases, the selection gradient vanishes and singular strategies are convergence‐stable. However, once adopted by the resident population, these singular strategies are evolutionarily neutral, indicating that mutant strategies have the same fitness as the resident population. The implications of fitness equality are common in classical game matrices, including the hawk–dove game. In the adaptive dynamics framework, such evolutionary neutrality represents a limit case between an ESS and an evolutionary branching point (Geritz et al., 1998). It has been shown that these points can turn into an invasion‐resistant strategy through only slight adjustments of the model structure (Dieckmann & Metz, 2006), and we will refer to them henceforth as ESSs.

Using this procedure, we explored how *β*_ESS_ varies with killing success (*u*_*i*_) by consecutively varying the killing success in each targeted life stage. Subsequently, we tested whether the killing of different life stages interactively influences *β*_ESS_ by simultaneously changing the killing success in all combinations of two different targeted life stages. In addition, we tested how interspecific killing changes the effects of male survival and mate competition parameters by varying killing success in each targeted life stage, while simultaneously varying fighter and scrambler survival (*P*_*f*_ and *P*_*s*_) or the mating competition parameters, *c*_*m*_ *, V, C* and *ε*, respectively. All of the parameters are changes with 100 steps within the value range of each parameter, as presented in Table 1.

### 2.5 Parameterizing the model for the bulb mite

From egg to adult, the bulb mite *R. robini* goes through a larval and two to three nymph stages. The life cycle takes at least 11 days if mites feed on a high‐quality food source (Smallegange, 2011). Adult males exhibit one of two ARTs: fighters or scramblers. Fighters have an enlarged third pair of legs that they can use to kill opponents with (Figure 1b). Fighters have also been observed killing conspecifics outside the context of direct competition for mates (Smallegange & Deere, 2014). Scramblers do not have an enlarged pair of legs, but mature faster and live longer than fighters (Smallegange, 2011) (Figure 1b). Male morph expression in the bulb mite is partly heritable and partly conditionally determined by final instar (tritonymph) size (Smallegange, 2011). Male final instars above a size threshold are more likely to mature into fighters; below the size threshold, they are more likely to mature into scramblers (Smallegange, 2011). All parameter values are taken from Smallegange and Johansson (2014), except values for *u*_i_ (*i* = *z*,* x*,* f*,* s*) that we set to zero as default, but in our model, analyses varied each value of *u* *i* between zero and unity (Table 1). In *R. robini*, the sex of individuals is determined by sex chromosomes (XX females, X0 males) (Oliver, 1977), thus, we assume no sex ratio adjustment by females (unlike, e.g., in Alonzo & Sinervo, 2001, 2007) and set *ρ* = 0.5. There are deviations in adults from a 1:1 sex ratio due to differences in life‐history trajectories between females and males (Smallegange, 2011), and due to adult fighters killing males (Smallegange & Deere, 2014). These differences and their effects on *β*_ESS_, however, are taken into account in our model as we take a life cycle approach.

## 3 RESULTS

**Figure 2:**
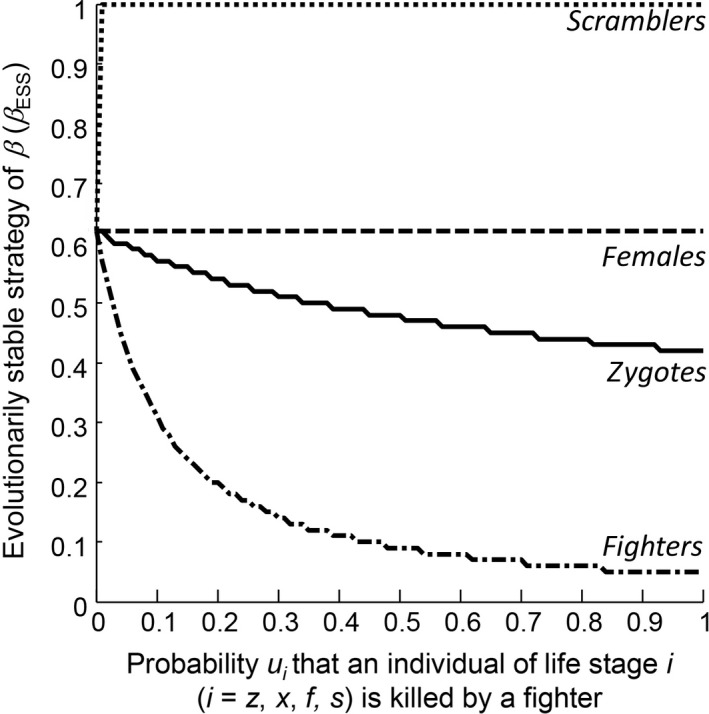
Effect of an increase in the probability *u*_*i*_ that an individual of each life stage (zygotes [solid line]; females [dashed line]; fighters [dot‐dashed line]; scramblers [dotted line]) is killed when encountered by a fighter on the evolutionarily stable strategy of *β* (*β* _ESS_). When fighters can kill scramblers, bistability occurs at very low values of *u*_*s*_

Firstly, we explored how the success of intraspecific killing by fighter males influences the ESS of *β*,* β*_ESS_. If the probability that a fighter kills a zygote (*u*_*z*_) or another fighter (*u*_*f*_) increases, the value of *β*_*ESS*_ decreases but is always higher than zero (Figure 2), indicating that fighters and scramblers always coexist if fighters kill zygotes or other fighters. The probability that a fighter kills a female (*u*_*x*_) has no influence on *β*_ESS_, and hence no effect on male morph coexistence (Figure 2). If the probability that a fighter kills a scrambler (*u*_*s*_) is very low (*u*_*s*_ < 0.005), evolutionary bistability occurs: *β*_ESS1_ is between 0 and 1 and increases with increasing *u*_*s*_, whereas *β*_ESS2_ = 1 (the value of *β* associated with the evolutionary repeller (*β*_*rep*_) in between *β*_ESS1_ and *β*_ESS2_ decreases with increasing values of *u*_*s*_; lower panels in Figure 4). At higher values of *u*_*s*_ (*u*_*s*_ > 0.005), *β*_ESS_ equals one (Figure 2). Therefore, if there is intraspecific killing of scramblers by fighters, males within populations are only fighters, but at very low probabilities of fighters killing scramblers, males within populations are either only fighters or a mixture of scramblers and fighters.

**Figure 3:**
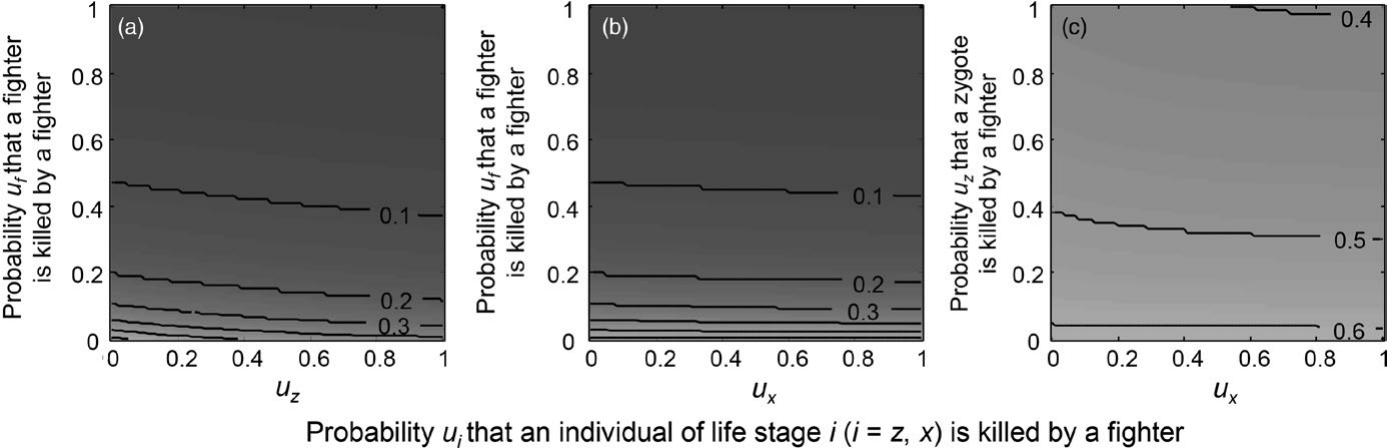
Effect of simultaneously changing two different probabilities of *u*_*i*_ (*i *= *z*,* x*,* f*) on the evolutionarily stable strategy of *β* (*β* _ESS_). Values of *β* _*ESS*_ range from zero (dark grey) to one (white); isoclines in each panel (black lines) denote equal values of *β* _*ESS*_ in increments of 0.1. Three different combinations of the effect of variation in two probabilities *u*_*i*_ on *β* _ESS_ are shown: *u*_*z*_ vs. *u*_*f*_ (a), *u*_*x*_ vs. *u*_*f*_ (b) and *u*_*x*_ vs. *u*_*z*_ (c)

Secondly, we explored how the intraspecific killing by fighters of individuals of two different life stages affects *β*_ESS_. For each value of *u*_*f*_, that is the probability that a fighter is killed when encountered by a fighter, *β*_ESS_ decreases with increasing values of *u*_*z*_ (probability that a zygote is killed when encountered by a fighter) (Figure 3a). Because the isoclines in Figure 3a (that denote equal values of *β*_ESS_) run parallel, we infer that there is no interactive effect between a simultaneous change in *u*_*f*_ and *u*_*z*_ on *β*_ESS_ (Figure 3a), that is the reduction in *β*_ESS_ with increasing values of *u*_*f*_ or *u*_z_ is independent of the success of killing individuals of the other life stage. In contrast, simultaneously changing values of *u*_*f*_ and *u*_x_ (probability that a female is killed when encountered by a fighter) interactively affects *β*_ESS_ (Figure 3b; inferred from the fact that the isoclines are not parallel). Similarly, simultaneously changing values of *u*_*z*_ and *u*_x_ also interactively affects *β*_ESS_ (Figure 3c; inferred from the fact that the isoclines are not parallel). It is interesting to note that variation in *u*_*x*_ alone does not affect *β*_ESS_ (Figure 2; dashed line); simultaneously varying *u*_*f*_ or *u*_*z*_ and *u*_*x*_, however, results in a decrease in *β*_ESS_ with increasing values of *u*_*x*_ (Figure 3b,c). It is noteworthy that in all of these comparisons, the evolutionary outcome is always male morph coexistence (Figure 3).

**Figure 4:**
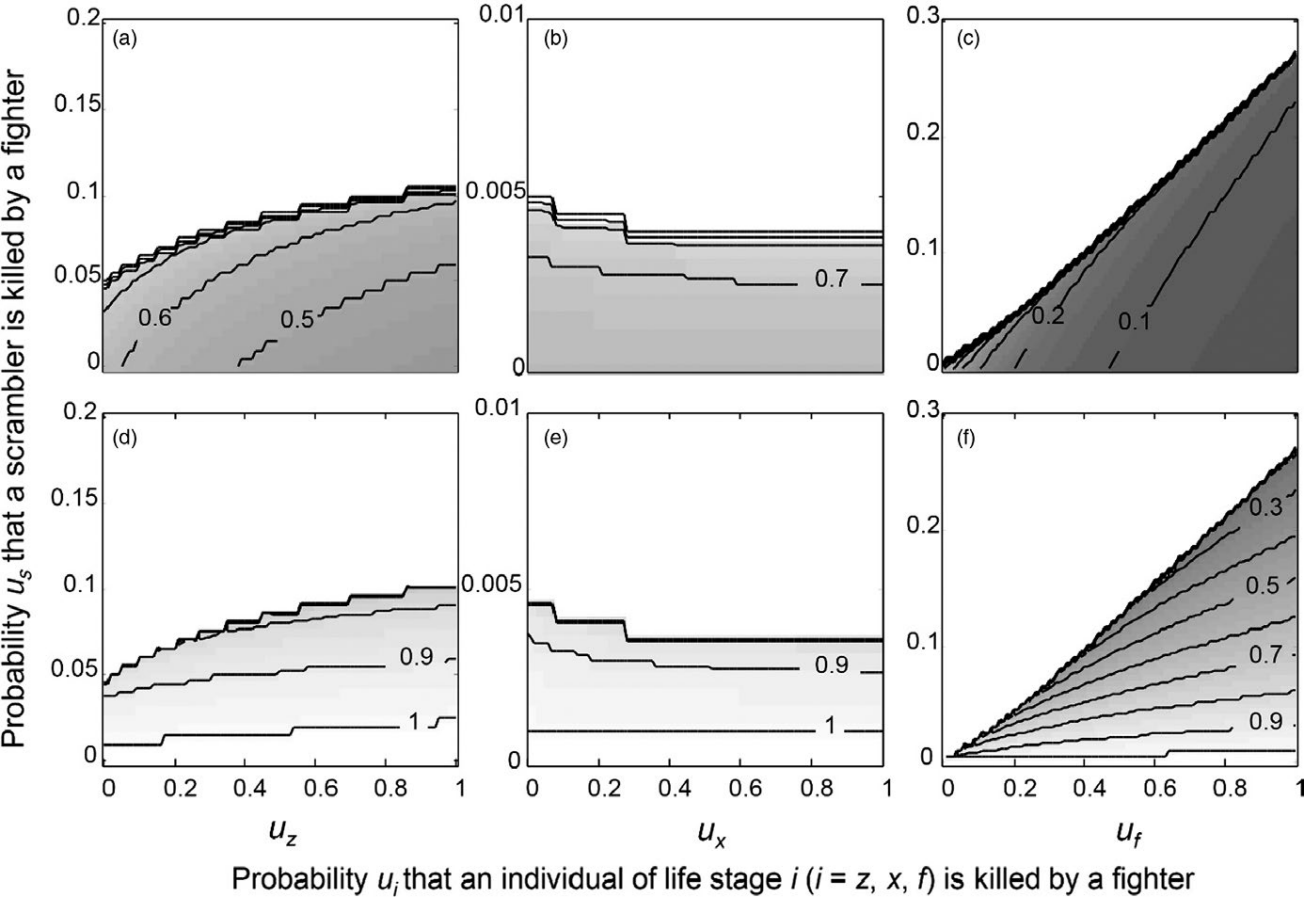
Effect of simultaneously changing the probability *u*_*s*_ that a scrambler is killed by a fighter, and either *u*_*z*_, *u*_*x*_ or *u*_*f*_ (where *z*,* x* and *f*, respectively, denote zygotes, females and fighters) on the evolutionarily stable strategy of *β* (*β* _ESS_). Values of *β* _*ESS*_ range from zero (dark grey) to one (white); isoclines in each panel (black lines) denote equal values of *β* _*ESS*_ in increments of 0.1. When varying *u*_*s*_, one of two evolutionary scenarios can occur: (a) a scenario with one evolutionarily stable strategy where *β* _ESS_ = 1; or (b) bistability, with two evolutionarily stable strategies of *β* (Figure 2; dotted line). The white area in each panel is associated with the first scenario. Within each panel, the non‐white area displays the bistability scenario where *β* _*ESS*__*1*_ and *β* _*ESS*__*2*_ are separated by a repeller. The value of *β* _*ESS*__*1*_ is shown in panels a–c, and the corresponding value of the associated repeller is shown in panels d–f. The value of *β* _*ESS*__*2*_ is always unity and not shown

The effect of simultaneously changing *u*_*s*_ (probability that a scrambler is killed when encountered by a fighter) with *u*_*z*_, *u*_*x*_ or *u*_*f*_ on *β*_ESS_ results in one of two evolutionary end scenarios occurring (Figure 2; dotted line): (a) a scenario with one ESS, where *β*_ESS_ = 1; or (b) bistability, with two evolutionarily stable strategies of *β*. The white areas in each panel of Figure 4 are associated with the first scenario and reveal that the range of values of *u*_*f*_ and *u*_*z*_ over which a single *β*_ESS_ = 1 exists increases with increasing values of *u*_*s*_ (Figure 4a,b,c). Male morph coexistence when fighters kill scramblers is therefore more likely to occur when fighters also target other fighters or zygotes. The non‐white areas in each panel of Figure 4 are associated with the second, bistability scenario, where *β*_ESS1_ and *β*_ESS2_ are separated by a repeller. The value of *β*_ESS2_ is always unity (not shown). The value of *β*_ESS1_ increases with increasing values of *u*_*s*_, and the increase is faster at higher values of *u*_*z*_ (Figure 4a) and *u*_*f*_ (Figure 4c; inferred from ever‐closer non‐parallel isoclines). In contrast, the value of *β*_ESS1_ decreases with increasing values of *u*_*s*_ for each value of *u*_*x*_ where bistability occurs (Figure 4b). The fact that the isoclines are parallel in Figure 4b suggests that the value of *u*_*x*_ does not affect the rate of change of *β*_ESS1_ with changing values of *u*_*s*_. Again, it is interesting to note that at nonnegative values of *u*_*s*_, the value of *β*_ESS1_ depends on the value of *u*_*x*_, in contrast to the situation when only *u*_*x*_ is varied and *u*_*s*_ = *u*_*z*_ = *u*_*f*_  = 0 (Figure 2; dashed line).

**Figure 5:**
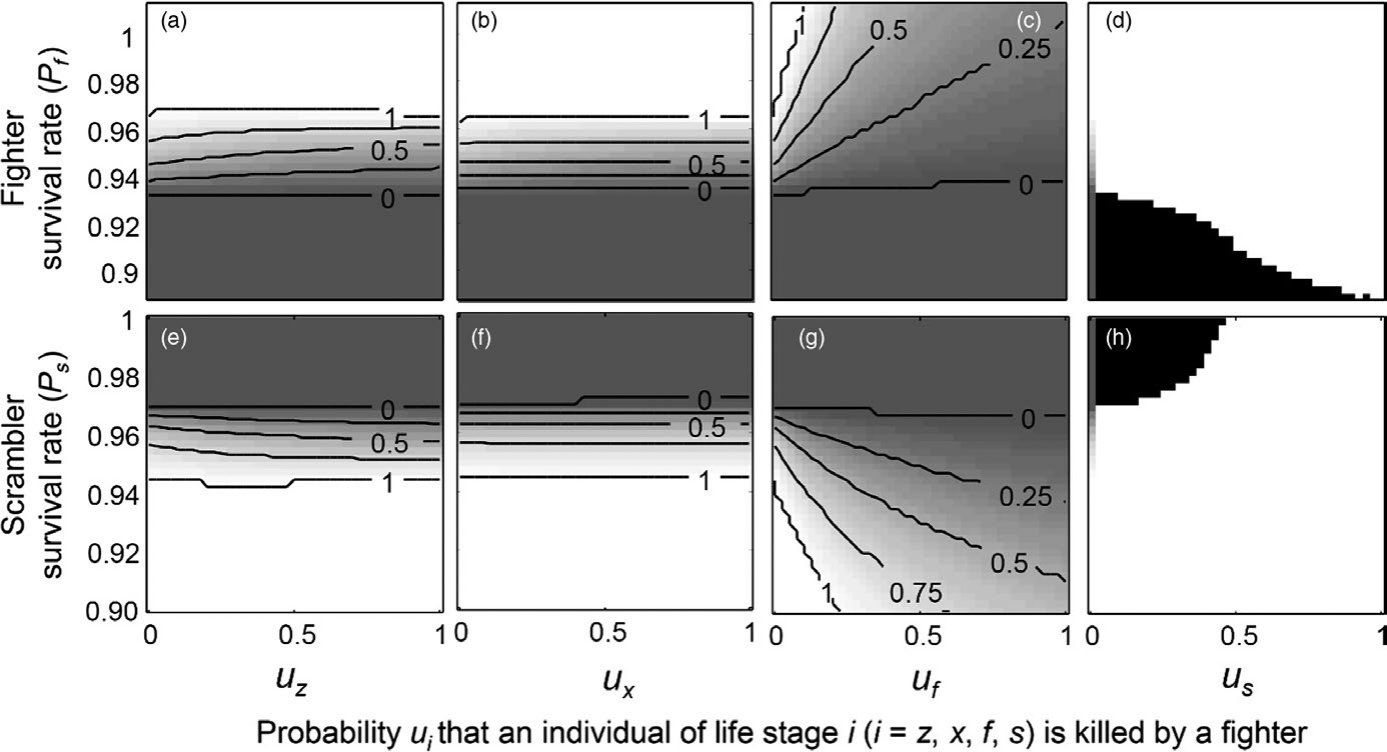
Effect of simultaneously varying the survival rate of fighters (*P*_*f*_) and the probability *u*_*i*_ that an individual of each life stage (zygotes (a), females (b), fighters (c) or scramblers (d)) is killed by a fighter on the evolutionarily stable strategy of *β* (*β* _ESS_); and the effect of simultaneously varying the survival rate of scramblers (*P*_*s*_) and the probability *u*_*i*_ that an individual of each life stage (zygotes (e), females (f), fighters (g) or scramblers (h)) is killed by a fighter on *β* _ESS_. Values of *β* _*ESS*_ range from zero (dark grey) to one (white); isoclines in panels (black lines) denote equal values of *β* _*ESS*_ in increments of 0.1. In panels (d) and (g), black areas indicate parameter combinations under which bistability occurs and an evolutionary repeller separates *β* _*ESS*__*1*_ = 0 and *β* _*ESS*__*2*_ = 1; in white areas in (d) and (g), *β* _ESS_ = 1

Thirdly, we explored the effects of simultaneously varying the probability *u*_*i*_ that an individual of life stage *i* (*i *= *z*,* x*,* f*,* s*) is killed by a fighter, and the survival rate of fighters (*P*_*f*_) or scramblers (*P*_*s*_) on *β*_ESS_. Across all values of *u*_*z*_ and *u*_*x*_, low fighter survival rates are associated with *β*_ESS_ = 0 and all males are scramblers; increasing fighter survival rate increases *β*_ESS_ until *β*_ESS_ equals unity and all males are fighters (Figure 5a,b). The range of survival rate values across which male morph coexistence occurs is relatively small (Figure 5a,b). A similar pattern is found at very low values of *u*_*f*_ for all values of *P*_*f*_ (Figure 5c); however, at higher values of *u*_*f*_, *β*_ESS_ decreases, but the rate of decrease strongly depends on *P*_*f*_ (Figure 5c). For nearly all parameter combinations of *u*_*f*_ and *P*_*f*_, *β*_ESS_ < 1, and both scramblers and fighters coexist (Figure 5c). When varying *u*_*s*_, male morph coexistence only occurs at the lowest values of *u*_*s*_ (Figures 2 and 5d); at higher values of *u*_*s*_, *β*_ESS_ is always unity, and all males are fighters (white area in Figure 5d) or bistability occurs (black region in Figure 5d), and all males are either scramblers (*β*_ESS1_ = 0) or fighters (*β*_ESS1_ = 1), depending on the starting conditions. The area of bistability decreases with increasing *u*_*s*_ (Figure 5d). We found similar responses but in the opposite direction when varying the survival rate of scramblers (*P*_*s*_) and the probability *u*_*i*_ that an individual of life stage *i* (*i *= *z*,* x*,* f*,* s*) is killed by a fighter (Figure 5e–h). High scrambler survival rates favour low values of *β*_ESS_ and low scrambler survival rates favour high values of *β*_ESS_; male morph coexistence occurs only over a small range of scrambler survival rate values, regardless of the value of *u*_*z*_ (Figure 5e) or *u*_*x*_ (Figure 5f). This pattern is again disrupted when varying *u*_*f*_, in which case the value of *β*_ESS_ decreases with increasing values of *u*_*f*_, but at different rates for different values of *P*_*s*_; as a result, male morph coexistence occurs across a greater range of scrambler survival rate values with increasing values of *u*_*f*_ (Figure 5 g). Again, when varying *u*_*s*_, male morph coexistence only occurs at the lowest values of *u*_*s*_ (Figures 2 and 5 h); at higher values of *u*_*s*_, *β*_ESS_ = 1, and all males are fighters (white area in Figure 5 h) or bistability occurs (black region in Figure 5d), and all males are either scramblers (*β*_ESS1_ = 0) or fighters (*β*_ESS1_ = 1), depending on the starting conditions. The area of bistability again decreases with increasing *u*_*s*_ (Figure 5 h).

**Figure 6:**
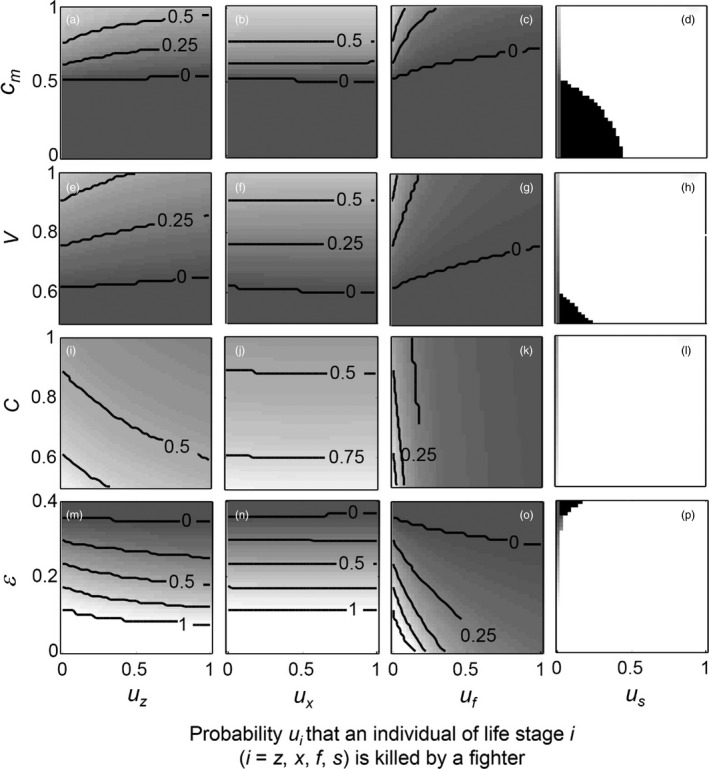
Effect of simultaneously varying one of the four competition parameters: *c*_*m*_, strength of male–male competition (a–d); *V*, probability of accessing a female without costs (e–h); *C*, costs of fighting against another fighter (i–l); and *ε*, probability of sneaking successfully when the opponent is a fighter (m–p), and the probability *u*_*i*_ that an individual of each life stage [zygotes (first column), females (second column), fighters (third column) or scramblers (fourth column)] is killed by a fighter on the evolutionarily stable strategy of *β* (*β* _ESS_). See further Figure 5

Finally, we explored the effects of simultaneously varying the probability *u*_*i*_ that an individual of life stage *i* (*i *= *z*,* x*,* f*,* s*) is killed by a fighter and varying each of the four competition parameters (*c*_*m*_ (strength of male–male competition), *V* (probability of accessing a female without costs), *C* (costs of fighting against another fighter) and *ε* (sneaker benefit)) on *β*_ESS_. Varying *u*_*z*_ and each of the competition parameters interactively affects the value of *β*_ESS_ (first column of Figure 6; interactive effects are inferred from non‐parallel isoclines in each panel), but the region of parameter space across which male morph coexistence occurs is only slightly affected, and only at low values of *u*_*z*_ and *ε* (Figure 6 m). Varying *u*_*x*_ and each of the competition parameters shows that *u*_*x*_ has little or no effect on *β*_ESS,_ and neither is the region of parameter space across which male morph coexistence occurs affected (second column of Figure 6). Varying *u*_*f*_ and each of the competition parameters interactively affects the value of *β*_ESS_, except in the case of the costs of fighting for fighter *C* (third column of Figure 6; interactive effects are inferred from non‐parallel isoclines). Only when varying *u*_*f*_ and *ε*, the sneaker advantage, is the region of parameter space across which male morph coexistence occurs affected; at the lowest values of these parameters, *β*_ESS_ = 1, and only fighters occur (Figure 6o). At higher levels of both parameters, the evolutionary endpoint is always male morph coexistence (Figure 6o). When varying *u*_*s*_ and each of the competition parameters, male morph coexistence occurs only at the very lowest values of *u*_*s*_, but for most other parameter value combinations, the evolutionary outcome is one where *β*_ESS_ = 1, or evolutionary bistability occurs and all males are either scramblers (*β*_ESS1_ = 0) or fighters (*β*_ESS1_ = 1), depending on the starting conditions (right‐hand column of Figure 6).

## 4 DISCUSSION

One of the key questions in evolutionary biology is: What maintains genetic and phenotypic variation? Prime examples of such diversity are male dimorphic species (Oliveira et al., 2008). Recent empirical results on the evolution and maintenance of ARTs emphasize how population feedback within an eco‐evolutionary feedback loop can influence the evolution of ARTs; that is, allowing for population feedback in experimental evolution studies on ART expression in the bulb mite shows that the observed direction of evolution of male morph expression can be different, and even diametrically opposed to the direction predicted by evolutionary theory (Smallegange & Deere, 2014). Negative population feedback is also the main driver of the expression of the genetically determined, alternative reproductive strategies of female side‐blotched lizards (*Uta stansburiana*) (Sinervo et al., 2000). Orange‐throated lizard females have few and large offspring, whereas yellow‐throated females have many and small offspring. In years of low population density, yellow‐throated females are favoured, but the many offspring cause an increase in population growth that overshoots carrying capacity. These new conditions favour orange‐throated females, and their offspring cause a decrease in population growth that undershoots carrying capacity. Again, only by taking the population feedback into account, can we understand the evolution and maintenance of these genetically determined, alternative morphs (Sinervo et al., 2000).

Here, we studied how the eco‐evolutionary interaction between evolution of ART expression and shifts in equilibrium population size and structure affects theoretical predictions of the ESS for male morph expression in the bulb mite, in relation to the extent to which fighters killed conspecifics of different life stages. We found that the killing of females had no effect on the evolution of male morph coexistence. This is probably because, although the killing of females reduces the number of reproducing females, each reproducing female produces larger clutches, as clutch size is only dependent on the number of females. In population‐dynamic equilibrium, larger clutch sizes therefore compensate for the smaller number of reproducing females. In reality, there will be a limit to the number of offspring that a female can produce in a single clutch, and when this is taken into account, we expect that the killing of females will affect fighter expression. Particularly, if there would be no (genetic) constraints on sex ratio expression (not in case of *R. robini* which has chromosomal sex determination), in which case ART coexistence depends on a complex interplay between ART frequency, female choice and sex allocation (Alonzo & Sinervo, 2001, 2007). Unsurprisingly, when fighters killed scramblers, the evolutionary endpoint was one in which males only matured into fighters, unless the killing rate was negligibly low, in which case we found bistability. In contrast, evolution favours male morph coexistence if fighters kill other fighters. This is in line with hawk–dove games where fighter costs are defined in terms of decreased survival, and which predict male morph coexistence when fighter costs are high relative to the potential rewards. Finally, when fighters targeted the juvenile zygotes, fighter expression decreased with increasing success at killing zygotes, probably because a reduction in zygote density reduces the long‐term population growth rate; hence, populations with fewer fighters have higher growth rates. Interestingly, this fighter cost is never outweighed by fighter benefits, because, regardless of the success rate of killing zygotes, evolution always favours male morph coexistence. The fitness costs incurred by fighters when they kill other fighters also carried over to increase the range of competition parameter values, and the range of scrambler and fighter survival rate values under male morph coexistence, and decreased the evolutionarily stable proportion of fighters in the male population. Our theoretical study into the evolution of ART expression not only highlights how the full eco‐evolutionary feedback loop affects ART expression, but also shows how the evolutionary outcome of male–male competition changes when details of this loop, like demography, intraspecific killing and competition, are incorporated into game‐theoretical analyses of male morph coexistence.

In our analyses, we assumed that the killing of conspecifics was not directly related to confrontations in male–male competition (as these costs are already incorporated into the competition payoff matrix **M**) but to other processes, the most likely one being cannibalism. Our results are therefore particularly relevant to systems in which the competitive (fighter) male morph not only uses its weaponry in male–male competition but also to cannibalize conspecifics. Victim mortality, energy extraction, size dependence and competition under cannibalism determine the population‐dynamic effect of cannibalism in the population (Claessen, de Roos, & Persson, 2004). Our model accounted for victim mortality by reducing the survival of the targeted life stage, and for competition by the incorporation of the competition payoff matrix. Size dependence was indirectly incorporated, because fighter males are generally a lot smaller compared to sneaker males and females (Smallegange, 2011). According to Claessen et al. (2004), the combination of these processes can lead to the stabilization of population dynamics when mortality weakens competition. We indeed found stable population dynamics when fighters killed zygotes or fighters. In addition, the combination of these processes can lead to evolutionary bistability when killing has an indirect positive effect on the cannibal, such as reduced competition. In our model, fighters directly reduce mating competition by killing scramblers, and we indeed found bistability when fighters killed scramblers. We did not account for any nutritional benefits that could be obtained from an intraspecific killing event. Therefore, to explore this scenario further, we adjusted the model such that intraspecific killing is now a cannibalistic event, and the energy gained from such an event is directly allocated to reproduction (Supporting information Appendix S1). This analysis shows that when fighters cannibalize other fighters, increased reproduction through cannibalism can compensate for the aforementioned negative fitness effects of the killing of other fighter males (Supporting information Figure S1). It is important to note that cannibalism only has a positive fitness effect on fighter expression if the gain in reproductive effort is greater than the reduction in population growth rate through the killing of fighter males. As, to our knowledge, it is unknown how much energy mites actually gain from feeding on conspecifics, we cannot compare this prediction against empirical data. In addition, it has been observed that bulb mites of other life stages feed on individuals killed by fighters (I.M. Smallegange, personal observation). To what extent this would affect the evolution of fighter expression, and hence male morph coexistence, remains to be explored.

Recently, a third male morph termed the mega‐scrambler has been identified in *R. robini* (Stewart, van den Beuken, Rhebergen, Deere, & Smallegange, 2018). Systems comprising three ARTs are not uncommon (Rowland & Emlen, 2009; Shuster & Sassaman, 1997; Sinervo & Lively, 1996), which raises the question of how the model could be extended to address three‐ART systems. Friedman and Sinervo (2016, Chapter 9) parameterized a Leslie matrix for elephant seals (*Mirounga angustirostris*) that includes three male ARTs that differ in reproductive success, and where males can switch between some, but not all tactics. Our model could similarly be extended to include the third male morph as another life stage. We do not yet know the life history of this third morph—whether it is an ontogenetic or post‐maturation, condition‐dependent transition, or perhaps genetically canalized—but a starting point would be to include into the model a threshold parameter, additional to *β*, to capture the proportion of scramblers that transition to the mega‐scrambler state. Rowland and Emlen (2009) found such two‐threshold mechanisms to occur in a clade of dung beetles where two developmental thresholds regulate the expression of horn size and form, yielding a facultative male trimorphism. Our model extended to three ARTs would allow for studying the evolution of male trimorphism in the context of conditional ARTs, possibly altering the ways in which we think about the evolution of conditional strategies.

In conclusion, this study shows that intraspecific killing can influence the evolution and maintenance of male ARTs. Interestingly, we showed that when fighters kill other fighters, this reduces their fitness and thus increases the relative fitness benefits of scrambler males. Perhaps in this way, selection can still favour scrambler expression, even if scramblers would suffer increased mortality, like Smallegange and Deere (2014) observed in their experimental evolution study, where selection against scramblers resulted in increased (and not decreased) scrambler expression. This “hidden” scrambler benefit could mean that ARTs are more likely to evolve in species with a similar life history. However, if fighters gain reproductive benefits from the killing of conspecifics through cannibalism, the detrimental effects on fighter fitness can be nullified. Alternatively, we have some evidence to suggest that maternal selection, that is the mother's phenotype affecting morph expression of her male offspring, could operate in bulb mites (Smallegange, 2011). In general, under certain conditions, such maternal effects on selection can cause a population to evolve maladaptively away from a fitness peak (Kirkpatrick & Lande, 1989), which could also explain the experimental evolution finding that selection against scramblers increased scrambler frequency (Smallegange & Deere, 2014). When investigating the evolution and maintenance of ARTs, one should therefore not only consider the effects of male–male competition and life‐history differences between phenotypes (cf. Smallegange & Johansson, 2014) or maternal selection, but also how the effects of population‐dynamical processes such as intraspecific killing and cannibalism feedback to influence the evolution of ARTs. The influence of such eco‐evolutionary feedbacks on trait evolution is not necessarily limited to polymorphisms like ARTs, but likely also plays a role in the evolution of other traits directly linked to demography, such as behavioural syndromes. The eco‐evolutionary feedback loop that we studied is one where ecology and evolution are played out at different time‐scales. Eco‐evolutionary interactions can also emerge when ecology and evolution occur on similar time‐scales (Sinervo et al., 2000), complicating the eco‐evolutionary process. But is the short‐term or the long‐term eco‐evolutionary selective process most decisive in the evolution of a trait? Short‐term, non‐adaptive plasticity can, for example, potentiate evolution by increasing the strength of directional selection (Chevin, Lande, & Mace, 2010), whereas short‐term adaptive plasticity can constrain long‐term evolution (Price, Qvarnström, & Irwin, 2003). Current empirical steps towards unravelling drivers of the eco‐evolutionary process (e.g. Sinervo et al., 2000), in concert with theoretical advances to model short‐term eco‐evolutionary processes (Coulson et al., 2017) that can be incorporated within the adaptive dynamics framework, pave the way for understanding how selection on different time‐scales affects trait evolution. This is particularly pertinent now with ongoing changes in the environment and climate, as eco‐evolutionary dynamics are most common during transient periods, for example, following an environmental change (Hiltunen & Becks, 2014).

## AUTHORS’ CONTRIBUTIONS

J.C.C., I.M.S. and M.E. conceived the ideas and designed methodology. J.C.C. carried out the analyses and led the writing of the manuscript. I.M.S. and M.E. contributed critically to the drafts and gave final approval for publication.

## DATA ACCESSIBILITY

MATLAB code is available in figshare: https://doi.org/10.6084/m9.figshare.3471941 (Croll, Egas, & Smallegange, 2018).
