# Supplementary material for: An eco‐evolutionary feedback loop between population dynamics and fighter expression affects the evolution of alternative reproductive tactics
Source: J Anim Ecol. 2018 Sep 19;88(1):11–23. doi: 10.1111/1365-2656.12899 (PMC7380021; doi:10.1111/1365-2656.12899)
Supplement: Supplementary file 1 [file JANE-88-11-s001.docx]

**Online Appendix: additional model extension to include reproduction gain through cannibalism.**

In the model, fighters gain no benefit from the killing of conspecifics but one obvious benefit is if the killing of conspecifics is considered to be cannibalism, in which case fighters would gain energy from the consumption of conspecifics. We can incorporate this energy gain into the model in the form of a higher survival rate or a higher fecundity. Here we chose to explore the effects of reproductive benefits arising from cannibalism, because this directly increases the fitness of the individual.

We assume that cannibalism of an individual from a specific life stage results in a fixed amount of additional energy. The number of offspring that can be produced with the amount of energy gained from cannibalizing one individual from life stage *i* (i = *z*, *x*, *f*, *s*) (Fig. 1, main text) is the conversion factor of that life stage (ω*_i_*). The total number of additional offspring of fighter males produced with energy from killing life stage *i (w_i_*), can then be calculated with $w_{i}=\omega_{i}q_{i}$, where q_i_ is the number of killed offspring from class *i,* as defined in the model in the main text.

To incorporate the additional offspring gained by cannibalism into the birth function, the fraction of gain, *b_f_*, has to be calculated by dividing the total number of additional offspring by the total number of offspring produced without cannibalism:

$b_{f}=\frac{\sum w_{i}}{B_{x,f}}=\frac{w_{z}+w_{x}+w_{f}+w_{s}}{B_{x,f}}$.

The fecundity of females and fighters with cannibalism by fighters can be calculated by multiplying the number of offspring (*B_x,j_*) with (1+ *b_f_*) resulting in respectively

$F_{f}=\frac{1}{2n_{f}}(1+b_{f})B_{x,f}$ ,

and

$F_{x}=\frac{(1+b_{f})B_{x,f}+B_{x,s}}{2n_{x}}$.

In this way, the energy gained from cannibalism is directed to reproduction but the path from consumption to additional offspring is not defined, because the number of offspring is increased directly. The number of offspring (*B_x,f_*) depends on the clutch size (*k*), the encounter rate of females and fighter males (*e_f,x_*) and the mating success of fighter males with females (*p_x,f_*). The energy gained by cannibalizing can be directed to all of these aspects, enlarging this parameter with a fraction (*b_f_*). When the energy gained from cannibalism is directed to reproduction through enlarging the clutch size (*k*), it is most likely that the clutch size in the absence of density-dependence (*k_0_*) is enlarged because, through cannibalism, the relative amount of food available to each individual enlarged. In this scenario, energy from cannibalism gained by the fighter male has to be transported to the female, to enlarge the clutch size. This is most likely to be done through a nuptial gift (Zeh & Smith, 1985). Fighter males can also use the additional energy gained from cannibalism to increase their encounter rate with females (*e_x,f_*). This can be done, for example, by more active searching behavior to find females.

The last option is one where the energy gained by cannibalizing is directed towards increasing the success of mating. It is most likely that, in that case, energy is used for something that enlarges the success of the mating in all situations (e.g. when competing against fighter males, competing against scrambler males or when no competition is present). Although the way in which energy from cannibalism is invested in mating is not defined, we can use the model to explore the overall effect of a benefit of cannibalism in the form of extra reproduction on the fraction of fighter males in the population. We did this by exploring the combined effect of increasing the success of cannibalism on a specific life stage (*u_i_*) and the additional reproduction gained by cannibalizing this life stage (*ω*_i_) on the fraction of fighter males in the population (*β*). When there is a gain of cannibalizing in the form of additional reproduction, this has a positive effect on the fitness of fighters, and results in a higher fraction of fighter males in the population, compared to a situation without gain from cannibalism (Fig. S1A). When cannibalism results in such high reproduction that outweigh any negative effects of cannibalism through reducing the population growth, cannibalism has positive fitness effects for fighter males. The same holds when fighters cannibalise zygotes (Fig. S1B), although, in that case, the reduction in population growth rate through the consumption of zygotes is not as great as when fighters consume other fighters; hence the a small gain in reproduction when consuming zygotes is required to outweigh the negative fitness effect of a reduced population growth rate. Cannibalism of females is directly profitable as soon as there is some sort of gain for the cannibalizing fighter males, because cannibalizing females does not have a negative effect on the population growth rate (Fig. S1C). Cannibalism of scrambler males already increases the fraction of fighters in the population. An additional gain for the cannibalizing fighter males increases this effect. In general, when cannibalism has an energy gain for fighter males, this increases the fitness of fighter males and makes it harder for scrambler males to be maintained in a population. Therefore, energy gain from cannibalism reduces the situations with cannibalism in which coexistence can occur.


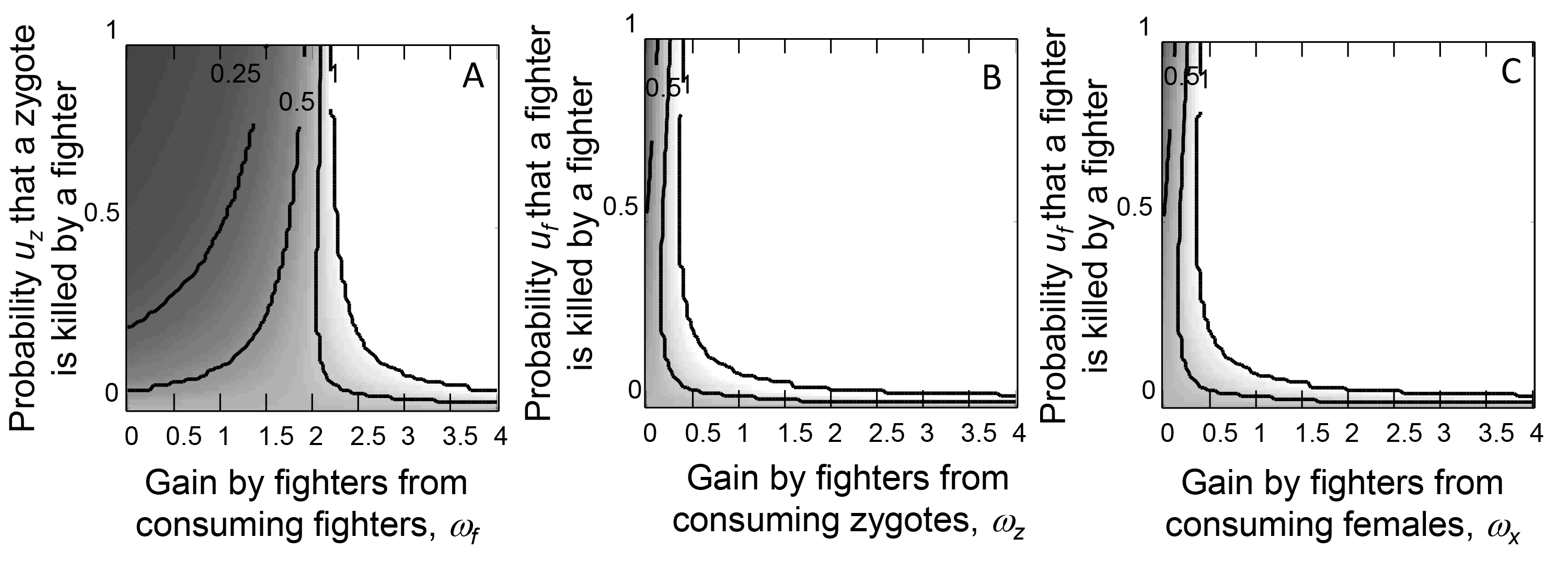


**FIGURE S1**

Effect of energy gain by fighters when they cannibalise other fighters (A), zygotes (B) or females (C) on the value of the evolutionairily stable strategy of *β* (*β_ESS_*). The value of *β_ESS_*  is depicted on a scale from dark gray to white with values between zero and one accordingly. Black lines are isoclines for every decimal to increase readability.

| **Table S1.** Parameter definitions and values for the model where juveniles have predetermined sex and morph. The definition and value of every additional parameter is given. When a parameter is varied in one of the analyses, the range in which this parameter is varied is also given. | | | | |
| --- | --- | --- | --- | --- |
| **Parameter** | **Definition** | **Value** | **Range when varied** | **Unit** |
| *Maturation* | | | | |
| *t_zx_* | Time to grow from zygote to female final instar | 10.1 |  | days |
| *t_zf_* | Time to grow from zygote to fighter final instar | 9.6 |  | days |
| *t_zs_* | Time to grow from zygote to scrambler final instar | 9.7 |  | days |
| *t_tx_* | Time to grow from final instar to adult female | 10.1 |  | days |
| *t_tf_* | Time to grow from final instar to adult fighter male | 9.6 |  | days |
| *t_ts_* | Time to grow from final instar to adult scrambler male | 9.7 |  | days |
| *Survival* | | | | |
| σ_tt_ | Survival rate of final instars | 1 |  | day^-1^ |
| *Intraspecific killing* | | | | |
| *u_tx_* | Probability that a female final instar is killed when encountered by a fighter male | 0 | .00-1.00 | - |
| *u_tf_* | Probability that a fighter final instar is killed when encountered by a fighter male | 0 | .00-1.00 | - |
| *u_ts_* | Probability that a scrambler final instar is killed when encountered by a fighter male | 0 | .00-1.00 | - |

**References**

Zeh, D. W., and R. L. Smith. 1985. Paternal investment by terrestrial arthropods. Am. Zool. 25:785-805.
